# Intraoperative Accidental Extubation during Thyroidectomy in a Known Difficult-Airway Patient: An Adult Simulation Case for Anesthesiology Residents

**DOI:** 10.3390/healthcare10102013

**Published:** 2022-10-12

**Authors:** David R. Okano, Javier A. Perez Toledo, Sally A. Mitchell, Johnny F. Cartwright, Christopher Moore, Tanna J. Boyer

**Affiliations:** 1 Department of Anesthesia, School of Medicine, Indiana University, Indianapolis, IN 46202, USA; 2 School of Medicine, Indiana University, Indianapolis, IN 46202, USA

**Keywords:** simulation, anesthesiology, difficult airway, accidental extubation, intraoperative

## Abstract

Intraoperative accidental extubation on a known difficult-airway patient requires prompt attention. A good understanding of the steps to re-establish the airway is critical, especially when the patient is known to have a difficult airway documented or discovered on induction or acquires a difficult airway secondary to intraoperative events. The situation becomes even more complicated if the case has been handed off to another anesthesiologist, where specific and detailed information may not have been conveyed. This simulation was designed to train first-year clinical anesthesia residents. It was a 50 min encounter that focused on the management of complete loss of an airway during a thyroidectomy on a known difficult-airway patient. The endotracheal tube dislodgement was simulated by deliberate tube manipulation through the cervical access window of the mannequin. Learners received a formative assessment of their performance during the debrief, and most of the residents met the educational objectives. Learners were asked to complete a survey of their experience, and the feedback was positive and constructive. The response rate was 68% (17/25). Our simulation program helped anesthesiology residents develop intraoperative emergency airway management skills in a safe environment, as well as foster communication skills among anesthesiologists and the surgery team.

## 1. Introduction

Complete loss of the airway during surgery is very dangerous and may have catastrophic consequences if not addressed promptly. The accidental removal of an endotracheal tube (ETT) may lead to temporary or permanent injury of the vocal cords if the ETT cuff is still inflated. It may also result in laryngospasms. After extubation, copious airway secretions may lead to aspiration and ultimately aspiration pneumonia. Unnoticed extubation may result in inadequate ventilation with possible sequelae of hypoxemia, hypotension, brain damage, cardiac arrest, and death [1,2,3,4].

This scenario has been developed to train the anesthesiology residents to experience causes of intraoperative endotracheal tube dislodgement that led to accidental extubation, steps to re-establish the airway on a known difficult-airway patient and communicate with the surgical team. The incidence of accidental extubation is not well documented in the OR setting, however, studies conducted in the intensive care unit (ICU) range from 0.5 to 35.8% in adults and 1% to 80.8% in the neonatal population [5,6,7,8]. Risk factors for a patient undergoing unplanned extubation in the ICU were: anatomy, high volume secretions, agitation, prone position, history of unplanned extubation, multiple transports between ICU and procedural areas, and frequent need for retaping the ETT [9]. While some of these would not apply to an OR setting, others should be considered. Regarding airway management, the incidence of difficult orotracheal intubation is 7.4% [10] and difficult mask ventilation is 7.8% [11]. Patient risk factors for difficult intubation include: snoring, retrognathia, micrognathia, macroglossia, short thick neck, BMI > 26 kg/m^2^, abnormal mandibular subluxation (SLUX) grade, Mallampati class [III/IV], Cormack Lehane grade [III/IV], abnormal atlantooccipital extension grading, flexion/extension deformity of neck, cervical spine abnormality, protuberant teeth, and mouth opening of less than 3 cm. Additionally, the experience level of the anesthesiologist/laryngoscopist is a risk factor [12].

When a surgical case is handed off intraoperatively to another anesthesiologist, it presents opportunities for various uncertainties. Even if the handoff was efficiently executed, it may not have been effective where specific and detailed information was not conveyed, such as the difficulty of mask ventilation and intubation, or at what depth the ETT was initially secured. Such uncertainties from the omission of information potentially delay the action to identify and solve the problem. We, therefore, reiterate the importance of effective communication during case hand-offs, including with surgical team members. This scenario was based on the author’s actual clinical experience.

## 2. Materials and Methods

The case is presented for facilitators in the Simulation Case file (Appendix A). The Simulation Case file consists of Simulation Case Overview (Table A1), Initial Presentation (Table A2), Instructor Notes–Changes and Case Branch Points (Table A3), and Instructor Notes–Ideal Scenario Flow and Anticipated Management Mistakes (Table A4). A critical actions checklist is included for learners to reference during the simulation session (Appendix B). Debriefing materials are included to facilitate the post-session (Appendix C).

### 2.1. Target Audience

The anesthesiology residency program at the Indiana University School of Medicine has 26 residents per cohort. We provide regularly scheduled simulation training of approximately four half-days per year per resident. A small group of three to five residents from the same cohort attend a session that consists of three, one-hour simulation scenarios. The contents range from procedural workshops and perioperative anesthetic management to mock objective structured clinical examination (OSCE) training. The accidental extubation scenario was introduced in 2020 and has been run yearly for each clinical anesthesia-year 1 (CA-1) residency cohort in approximately their ninth week of training. One simulation faculty has served as the primary facilitator for running and iteratively updating this scenario.

### 2.2. Equipment and Environment

We aimed for the highest levels of fidelity and realism. Participants were provided proper surgical attire for each role, such as scrubs, masks, hats, gloves, patient gowns, and surgical gowns. The OR could be either a real (in situ) or mock/simulated laboratory. This simulation occurred in a mock OR setting. The mannequin, operating table, monitors, and equipment (a fully equipped anesthesia cart with various sizes of supraglottic airways (Laryngeal Mask Airway^®^ or i-gel^®^), video laryngoscope, stethoscope, and a standard set of simulated anesthesia drugs) were in the OR. A code cart was available outside of the OR. The observation, facilitation, and programming of the scenario were conducted via wireless audio and video feed from the mock OR to a separate control room that was concealed from the learners.

**Figure 1:**
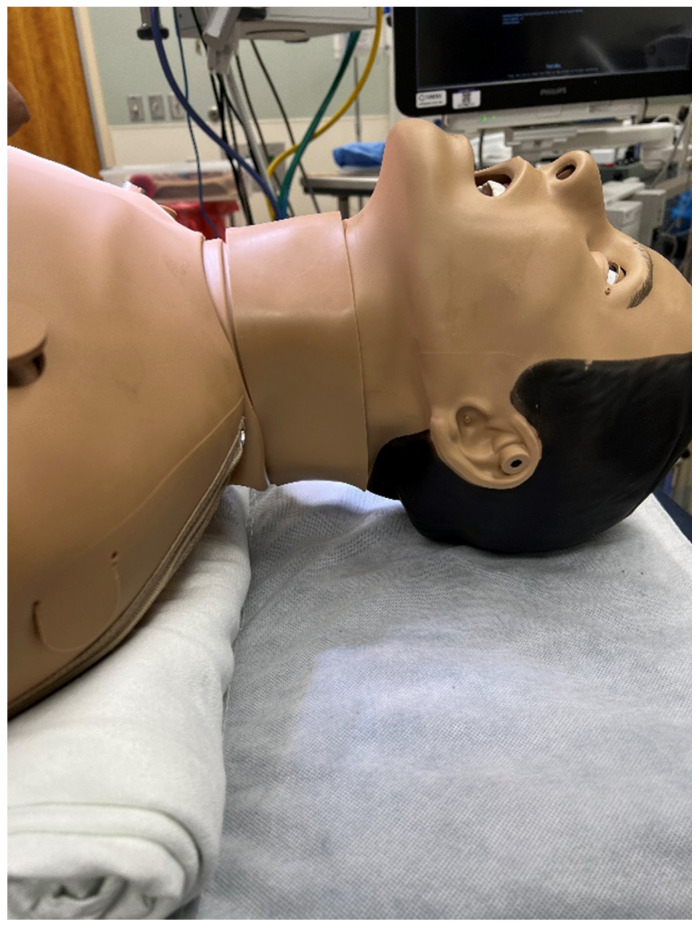
Mannequin with a shoulder roll with its neck extended.

**Figure 2:**
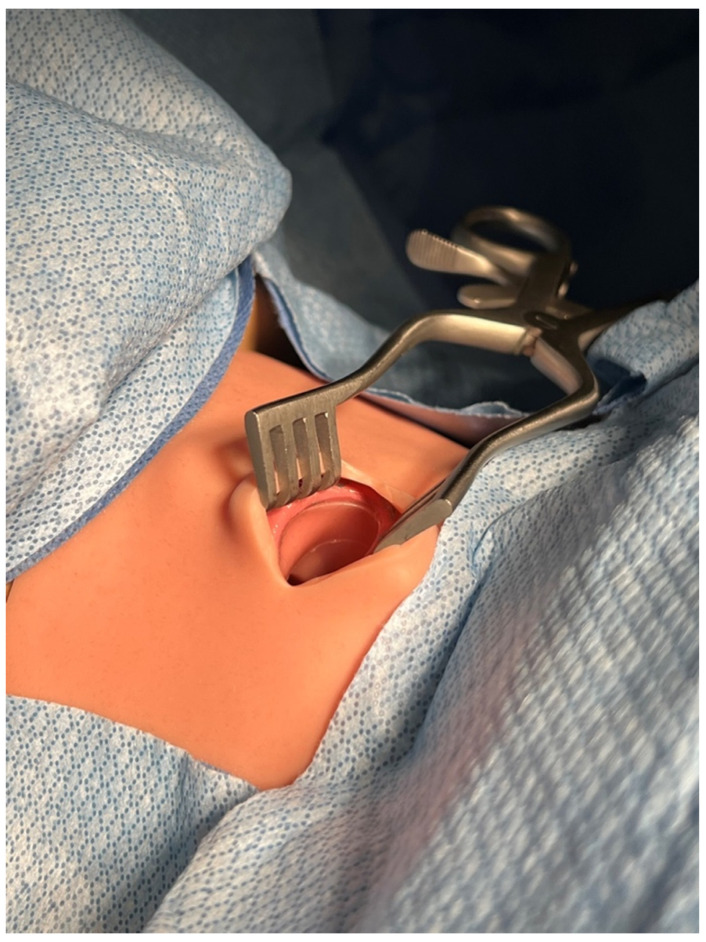
Opened access window with a retractor.

**Figure 3:**
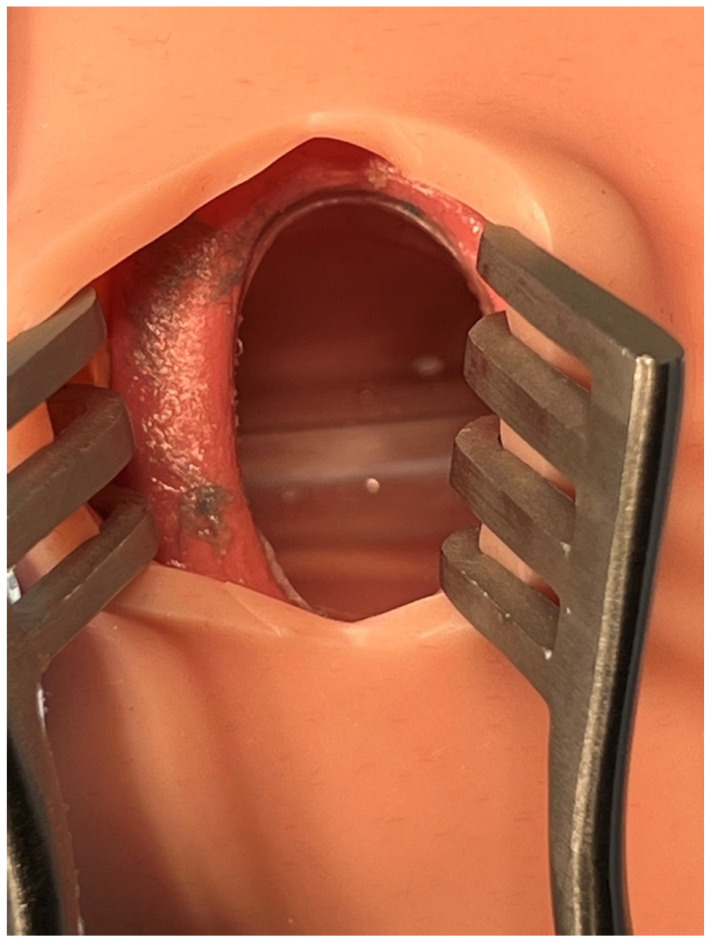
Endotracheal tube is seen in the trachea.

We used a human-patient simulator mannequin (Laerdal SimMan 3G+), a high-fidelity lung simulator (IngMar ASL 5000 Lung Solution), an interconnected software interface (LLEAP), and a decommissioned yet fully functioning anesthesia machine (Dräger Apollo). An EKG monitor, blood pressure cuff, and pulse oximeter were connected, and the vital sign data were displayed on the simulated patient monitor. The mannequin was positioned supine on the operating table with one peripheral IV in the upper extremity, intubated, and on the anesthesia machine ventilator. A shoulder roll was placed under the mannequin and the neck in full extension (Figure 1). A surgical drape covered the mannequin from head to toe with a fenestration at the anterior cervical region as the surgical field. The edges of the surgical drape near the head were raised and attached to IV poles for the anesthesiologist’s ease of access to the head. The seal on the anterior cervical airway access window was removed to enable manipulation of the ETT. The window was covered by silicone skin with a small incision that was held open by a surgical retractor (Figure 2 and Figure 3).

### 2.3. Personnel

Role assignments for the simulation were flexible depending on the number of participants. The minimum requirements for this scenario were: one learner anesthesiologist (target learner) who takes over the case, one anesthesiologist (embedded participant, EP) who hands off the case to the learner, and one surgeon EP. Additional residents could be EP surgical scrubs or circulator nurses. Small groups of three to four anesthesiology residents participated in the scenario. One resident was in the “hot seat” as the learner anesthesiologist and the others were assigned EP roles. Faculty members and medical students also served as simulated participants as needed. Simulation operation specialists programmed and operated the mannequin and simulated monitors. The faculty facilitator was also in the control room during the simulation and gave verbal cues and instructions through headsets worn by the EPs.

### 2.4. Implementation

The EP anesthesiologist was prebriefed (Appendix D) and instructed on which verbal information to provide the learner anesthesiologist during handoff. The EP surgeon was prebriefed (Appendix E), instructed to wear the headset, and wait in the OR. The learner anesthesiologist was instructed to enter the OR and take over the case in progress.

The scenario started with the patient intubated, under general anesthesia, as the surgeon worked on the total thyroidectomy. The patient’s history was given as morbidly obese, with a large neck circumference, and a known difficult airway. The EP anesthesiologist told the learner anesthesiologist that “this patient was difficult to mask ventilate due to his beard and big tongue, and the intubation was accomplished by utilizing a video laryngoscope”. The anesthesiologist mentioned a minor ETT leak was heard from the patient’s oral cavity, and the issue was resolved after adding 10 mL of air to the cuff.

**Figure 4:**
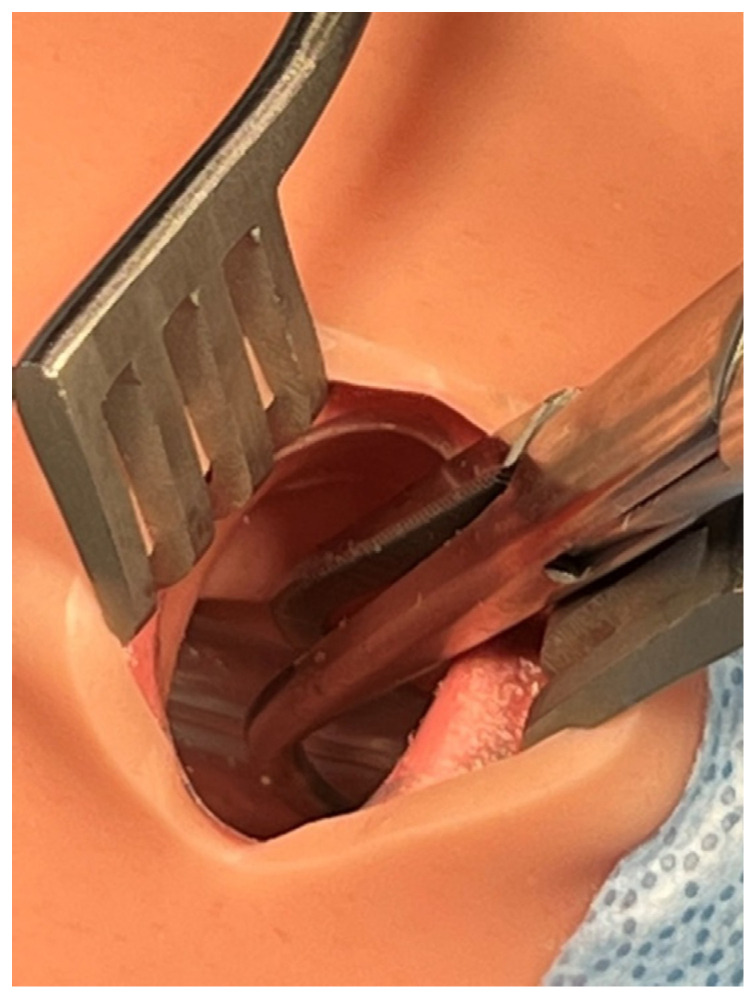
Endotracheal tube grasped by Kelly forceps.

The facilitator instructed the EP surgeon to mimic accidental extubation by deliberately pushing out the endotracheal tube from the trachea (Figure 4). Kelley forceps were inserted through the access window of the anterior neck of the mannequin to accomplish this task. This manipulation by the surgeon was solely to mimic accidental extubation and was not intended to reproduce a surgical breach of the airway. The surgeon denied allegations from the anesthesiologist whether they have damaged the trachea or the ETT, and physically blocked the view of the surgical site from the anesthesiologist.

**Figure 5:**
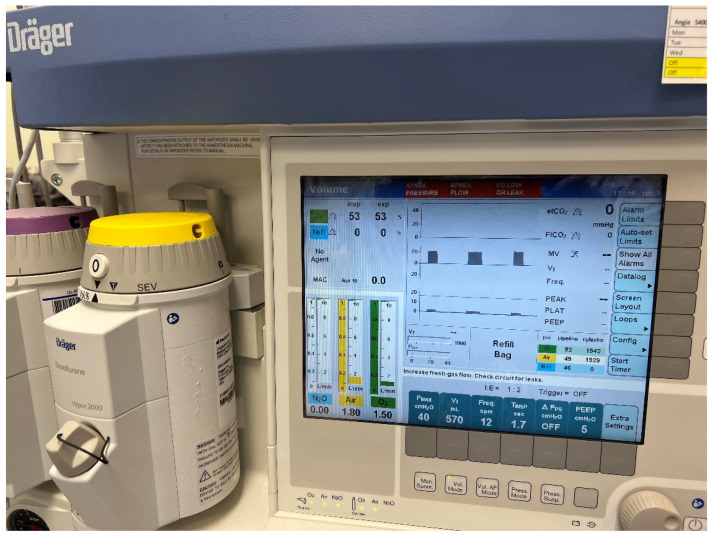
Anesthesia ventilator displaying alarms indicating total loss of airway.

**Figure 6:**
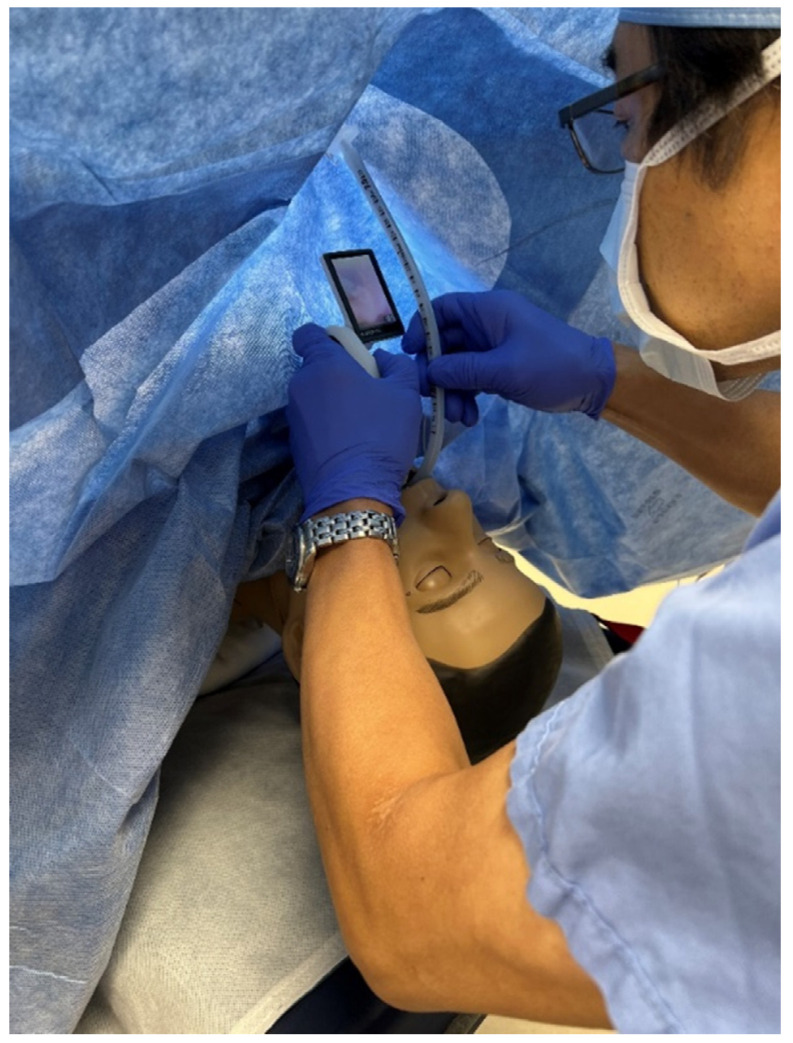
The anesthesiologist reintubating under the drape with a video laryngoscope.

The learner anesthesiologist heard the cuff leak sound as the ETT was dislodged by the surgeon. As they tried to troubleshoot, the ventilator alarm sounded, and the bellows completely collapsed (Figure 5). The anesthesiologist should have realized the ETT was dislodged and immediately notified the surgical team. Attempts to mask ventilate failed, and the patient desaturated. Having known the patient has a difficult airway, the anesthesiologist should have planned the steps to quickly re-establish the airway with a supraglottic airway device and then reintubate with a video laryngoscope (Figure 6).

The scenario ended when the anesthesiologist successfully re-established the airway and communicated with the surgeon to resume the surgery. We found that the simulation took approximately 50 min, including the prebriefing of the participants.

### 2.5. Assessment

The facilitator reviewed the completion of the Critical Actions Checklist (Appendix B). Learners received formative feedback during the debriefing.

### 2.6. Debriefing

The debriefing was held immediately following the conclusion of the scenario and lasted approximately 20 min. All the participants, including observers, relocated to a nearby classroom; alternatively, they could have remained in the mock OR for the debriefing. The debriefing session began with an open-ended question to the learner who was in the “hot seat” about how they felt the scenario went. This is a strong tool to elicit spontaneous reflections from the learner as well as stimulate active discussions from the other participants. The following elements and talking points are recommended during the debriefing with the learners.

**Figure 7:**
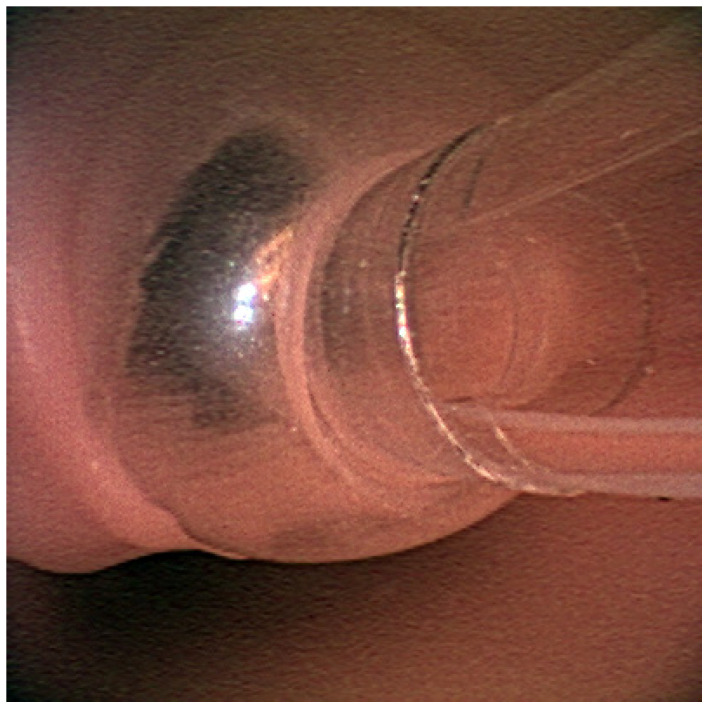
View from the oral cavity. Partial herniation of the endotracheal tube cuff from the glottis.

**Figure 8:**
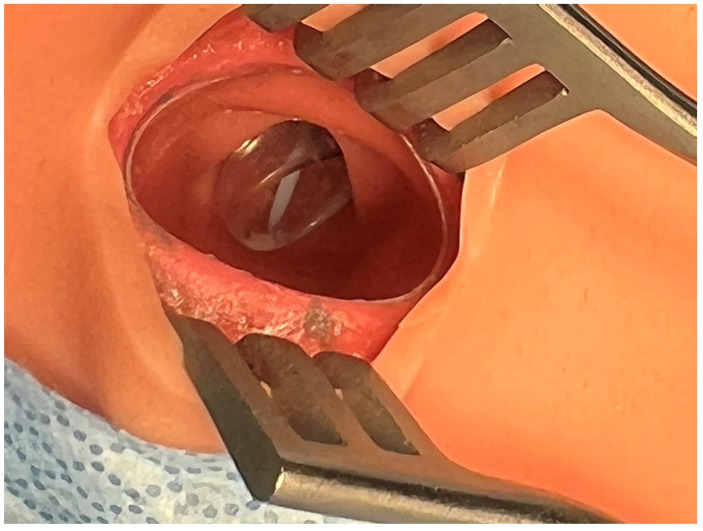
View from inside the access window as the distal end of the endotracheal tube barely remains in the glottis.

**Figure 9:**
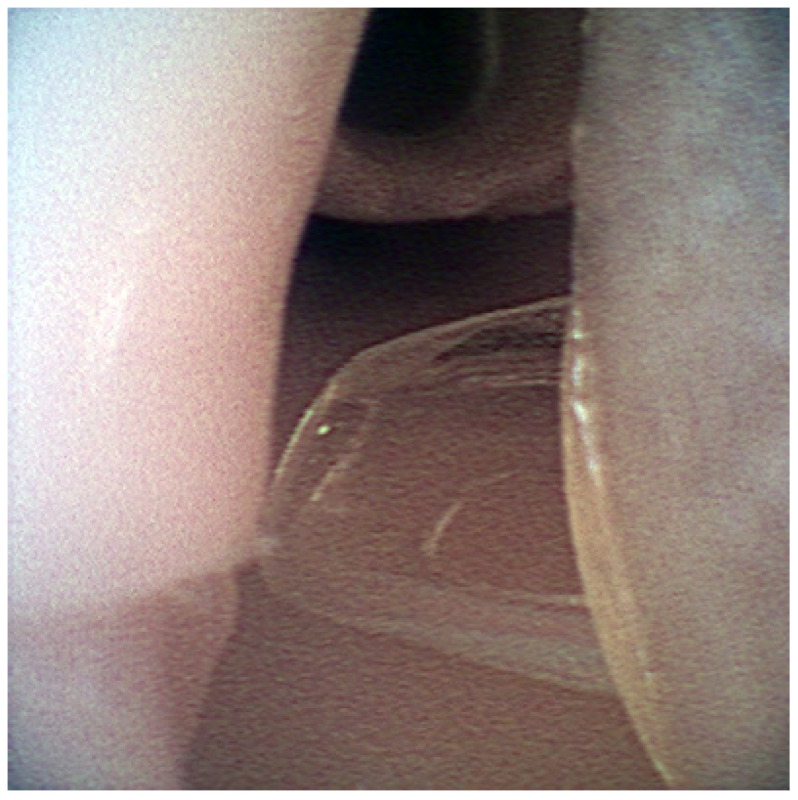
View from the oral cavity. Complete dislodgement of the endotracheal tube.

1 Communication between the anesthesiologists handing off and taking over: It is a common practice to sign out an ongoing case to another anesthesiologist. Because the handoff takes place in a busy operating room setting, it is common for there to be frequent interruptions. The learner anesthesiologist should discuss how to perform handoffs efficiently and without omissions. Were there omissions of any critical information? What did the anesthesiologist taking over assume when they were informed of a leak that was resolved by adding 10 mL of air to the ETT cuff? Did both anesthesiologists attempt to discuss or explore the root cause?
2 The cause of accidental extubation: The facilitator lists the three factors that contributed to the migration of the endotracheal tube and resulted in accidental extubation. The learners discuss these factors:
  - Maximum cervical extension: Although the endotracheal tube was initially taped at the correct depth, it was pulled out with cervical extension and the cuff partially herniated out of the vocal cords enough to create an air leak (Figure 7).
  - Adding more cuff air to seal the leak: As more cuff air was added to remedy the leak without repositioning the endotracheal tube, the tube continued to slide out although the air leak seemed to have temporarily resolved. Only the distal end of the endotracheal tube remained beyond the vocal cords while the cuff was inflated with more than 20 mL of air (Figure 8).
  - Surgical manipulation of the neck: The vibration and movement transferred from the surgical manipulation of the neck dislodged the endotracheal tube completely from the vocal cords (Figure 9).
3 Recognize accidental extubation: Learners discuss what alarms they received when the integrity of the airway was lost. What steps should the learner take to isolate the problem and discover the accidental extubation?
4 Communication with the surgical team: Emphasize the importance of notifying the loss of the secure airway and asking for help. Should they immediately activate Code Blue in this situation?
5 Re-establishment of the secure airway: Learners discuss the steps and the potential obstacles in re-establishing oxygenation and ventilation on a rapidly de-saturating patient with a known difficult airway.
  - Initiate bag-mask ventilation: Obstacles are secretions, facial hair, large tongue, maximum cervical extension, head distance from the bag and anesthesia machine, and field avoidance.
  - Insert supraglottic airway: Obstacles are unavailability of the device, incorrect device size, and large leak from poor seal due to anatomy and/or size.
  - Draw up drugs required for reintubation: Obstacles are drugs not immediately available in the OR, no one to help draw up drugs.
  - Reintubate: Obstacles are known difficult airway, maximum cervical extension, inexperienced laryngoscopist, unavailability of equipment: video laryngoscope, difficult airway cart, adjunct devices (i.e., bougie, Eschmann, Frova, Aintree, fiberoptic/video bronchoscope).

## 3. Results

All simulation sessions involved formative learning and assessment to benefit the residents. The residents met the educational objectives and performed very well by promptly recognizing the accidental extubation and communicating with the surgical team. They quickly attempted mask ventilation and once that failed, they utilized a video laryngoscope to reintubate the known difficult-airway patient. The facilitator redirected the learner anesthesiologists by giving verbal cues to the EP surgeon via a microphone and earpiece.

Residents were asked to provide feedback at the end of each session. A QR code linked to the online survey was posted on the wall of the debriefing room, and email reminders were sent with the survey link to increase the response rate. The overall feedback from our learners was positive with a survey response rate of 68% (17/25) for AY 21–22 (Table 1). Constructive comments will be used for iterative improvements.

## 4. Discussion

The purpose of the development of this simulation was to enable residents to practice crisis management skills for potential perioperative incidents as well as to foster excellent communication skills with patients and the perioperative team as mature anesthesiologists. We endeavored to provide a psychologically safe learning environment, from scenario design, the prebrief with the basic assumption [13], scenario design, and debriefing exercises facilitated by trained faculty members. This empowered residents to make and learn from their mistakes and the mistakes of others. Additionally, the faculty were able to address misconceptions and knowledge gaps.

Although the endotracheal tube was secured at the proper depth immediately after intubation, it may become displaced due to subsequent positional changes, surgical manipulation, or loosening of the tape securing the tube. Since this case was a thyroidectomy, the patient’s neck was maximally extended with a shoulder roll. One report showed that an ETT can be displaced cephalad by approximately 7 to 20 mm with full neck extension [14]. The degree of tube displacement increases in patients with larger body mass index and those with shorter neck lengths [15]. When the endotracheal tube cuff begins to herniate from the glottis, a leak will start to occur, and overinflating the cuff to correct the leak causes the cuff to herniate further and temporarily decreases the leak. Eventually, the pressure and vibration from the manipulation in the surgical field cause complete dislodgement of the endotracheal tube. To reproduce this sequence of events on a mannequin, we devised a new technique in which an endotracheal tube is grasped with Kelly forceps or similar instruments through an access window located on the anterior of the neck of SimMan3, and then the tube is gradually pushed out through the trachea into the oral cavity.

Once the learner anesthesiologist realized that the endotracheal tube had been accidentally extubated, they were forced to re-establish ventilation. However, the mannequin was programmed to be very difficult to mask ventilate. With the patient rapidly desaturating, there was not enough time to draw up propofol, muscle relaxants, or prepare a video laryngoscope, and the airway had to be temporarily secured with a supraglottic airway. The SimMan3 mannequin is not suitable for insertion of a supraglottic airway, and this becomes a limitation of this simulation. Even with adequate lubrication, it is difficult to insert the supraglottic airway into this mannequin to the proper depth and achieve an adequate seal. Therefore, when residents attempted to insert the supraglottic airway, even incomplete insertion should be considered as a re-establishment of ventilation. To make it evident, we displayed ETCO_2_ and improved oxygen saturation on the patient monitor.

Due to time constraints, one of the limitations of this simulation was that only one or two residents from each small group were placed in the “hot seat” as the learner anesthesiologist. Although feedback from the other residents indicated that they also learned from the simulation, it remains to be seen if there is a difference in the effectiveness of training between the residents in the “hot seat” and those in the other roles. Exploring these questions and possible scenario adjustments are the subjects of our future research.

One of the reasons for the low response rate on the residents’ survey (68%) was that we did not make the residents finish the survey immediately after the completion of the debriefings since they all had to resume their regular clinical anesthesia tasks promptly. It is well known that the debriefing should occur immediately after the simulation since the thoughts and feelings will fade fairly quickly [16]. A similar principle should be applied to the post-simulation survey. Providing learners with dedicated time prior to leaving the simulation center to complete an online survey as opposed to having them complete it sometime later without a solid deadline would increase response rates in the future.

## Figures and Tables

**Table 1:** Distribution of responses, item mean scores, and standard deviation.

Item ^a^	Percentage (n)				
	Agree ^b^	Neutral ^c^	Disagree ^d^	M	SD
1. Before this simulation session, I could confidently identify intraoperative Accidental Extubation.	82(14)	12(2)	6(1)	2.06	0.73
2. Before this simulation session, I could confidently explain the mechanism of tube migration and Accidental Extubation during Thyroidectomy.	70(12)	24(4)	6(1)	2.12	0.83
3. Before this simulation session, I could confidently re-establish a secure airway on a quickly desaturating, known difficult-airway patient.	64(11)	24(4)	12(2)	2.18	0.98
4. My confidence in how to identify intraoperative Accidental Extubation has improved as a result of this simulation session.	94(16)	6(1)	0	1.29	0.57
5. My understanding of the mechanism of tube migration and Accidental Extubation during Thyroidectomy has improved as a result of this simulation session.	100(17)	0	0	1.24	0.42
6. My confidence in how to re-establish a secure airway on a quickly desaturating, known difficult-airway patient has improved as a result of this simulation session.	94(16)	6(1)	0	1.29	0.57
7. The debriefing faculty created a psychologically safe learning environment throughout the debriefing session.	100(17)	0	0	1.06	0.24
8. I received useful feedback and the most important issues were summarized during the debriefing sessions.	100(17)	0	0	1.24	0.42
9. I had the opportunity to ask questions during the debriefing session.	100(17)	0	0	1.06	0.24

^a^ Rated on a 7-point Likert scale (1 = strongly agree, 2 = agree, 3 = somewhat agree, 4 = neutral, 5 = somewhat disagree, 6 = disagree, 7 = strongly disagree). ^b^ Strongly agree, agree. ^c^ Somewhat agree, neutral, somewhat disagree. ^d^ Disagree, strongly disagree.

## Data Availability

The evaluation forms electronically filled by the participating residents are stored on Indiana University Qualtrics website. The website is not open to the public. Please contact the corresponding author for access to survey data.

## Appendix A. Simulation Case

**Table A1:** Simulation Case Overview.

SIMULATION CASE TITLE: Intraoperative Accidental Extubation during Thyroidectomy in a Known Difficult-Airway Patient: An Adult Simulation Case for Anesthesia Residents	
**Brief narrative description of case**	A morbidly obese, known difficult-airway patient is undergoing total thyroidectomy. The learner anesthesiologist takes over the case from the other anesthesiologist. Shortly after taking over the case, the endotracheal tube slides out, causing complete loss of airway to occur. The patient quickly starts to desaturate, and the ventilation needs to be re-established as soon as possible. This scenario was developed to train the anesthesia residents to understand the steps to reestablish ventilation in accidental intraoperative extubation on a known difficult-airway patient. The exercise will also reiterate the importance of efficient communication during case hand-offs between anesthesiologists.
**Primary Learning Objectives**	Perform handoffs between anesthesiologists efficiently and without omissions upon changeover. Understand the mechanism of intraoperative endotracheal tube dislodgement leading to accidental extubation. Diagnose intraoperative accidental extubation. Understand the steps to re-establish a secure airway on a quickly desaturating known difficult-airway patient. Demonstrate effective communication with surgeons and the surgical team in the operating room (OR).
**Critical Actions**	Take over the ongoing thyroidectomy case from another anesthesiologist. Acknowledge the critical information regarding the patient’s difficult airway mentioned during the hand-off. Recognize the recurrence of cuff air leak and take action to fix the problem. Recognize the ventilator alarms indicating a complete loss of airway. Identify the cause of the complete loss of airway as accidental extubation. Alert the surgeon and the surgical team in the OR. Remove the extubated endotracheal tube, shut off the vaporizer for the volatile anesthetic, clean the patient’s airway of secretions, and attempt bag-mask ventilation. Ask the surgeon to remove the shoulder roll if necessary. Recognize the quick desaturation of the patient. Ask for more help or call Code Blue. After failing the attempt of bag-mask ventilation, insert a supraglottic airway to restore the ventilation temporarily. Prepare necessary medications for reintubation (Propofol and Succinylcholine). Reintubate the patient with a video laryngoscope. Communicate with the surgeon that the airway has been re-established and the patient is ready to resume the surgery.
**Learner Preparation or Prework**	This is a 40 min small group simulation scenario designed for CA-1 anesthesia residents who are typically 8 to 9 weeks into the residency training. They are expected to be competent at inserting supraglottic airways (Laryngeal Mask Airway^®^ or i-gel^®^), endotracheal intubations of easy to moderately difficult airways, with additional knowledge of rapid sequence induction. The learner anesthesiologist will NOT receive any stem or a description of the patient before entering the OR since another anesthesiologist will verbally deliver that information during the hand-off.

**Table A2:** Initial Presentation.

Initial Presentation PATIENT NAME: Nick McNoneck PATIENT AGE: 51 CHIEF COMPLAINT: Thyroid Goiter PHYSICAL SETTING: Operating Room			
**Initial vital signs**	BP 127/83 mmHg, HR 76, Sinus rhythm, RR 10 on Volume Controlled Ventilation, SpO_2_ 97% on FiO_2_ 40%, T 36.8 °C		
**Overall Setting and Appearance**	The OR is a room that is designed to appear as an OR. The learner will find the mannequin already intubated and under general anesthesia, lying supine on the OR table, hooked up to IVs, and monitors, and already prepped and draped. A shoulder roll is under the patient, and the mannequin’s neck has been fully extended. There is an anesthesiologist in the room who will change over the case to the learner. A surgeon is fully scrubbed in, and the thyroidectomy has already been going on. A fully equipped anesthesia cart loaded with various sizes of supraglottic airways, a video laryngoscope, a stethoscope, and other medicines required for anesthesia is placed right next to the anesthesia machine. A code cart is available in the hallway outside the OR.		
**Standardized Participants and their roles in the room at case start**	The facilitator will observe the progress of the scenario from outside the room in both scenes via half-mirror or audiovisual feed. **The Leaving Anesthesiologist:** Played by the other anesthesia resident in the small group. In the scenario, this anesthesiologist has started the case and provides hand-off to the learner anesthesiologist who is taking over the case. “Hi, I need to leave early today. I will sign this case out to you”. **Surgeon:** Played by the other anesthesia resident in the small group. The surgeon will be fully scrubbed in and is performing the thyroidectomy. The surgeon is instructed by the facilitator to gain access to the endotracheal tube via the access window on the mannequin’s neck. **Another surgeon:** Played by the other anesthesia resident who initially played the role of the anesthesiologist. After the changeover, he/she changes the role, gets fully scrubbed in, and participates in the surgery. **Circulator nurse:** Played by another anesthesia resident in the small group.		
**History of Present Illness (HPI)**	The patient is a 51-year-old male with a thyroid goiter scheduled for a total thyroidectomy. The patient endorses some difficulty swallowing. He denies difficulty breathing but usually sleeps with his head elevated. The patient is 186 cm tall and weighs 183 kg. The surgeon has requested the anesthesiologists not to use long-acting paralytics after the induction since he/she plans to monitor the recurrent laryngeal nerve during the surgery.		
**Past Medical/Surgical History**	**Medications**	**Allergies**	**Family History**
Morbid obesity (BMI 53) Hypertension Hyperlipidemia Obstructive Sleep Apnea Gastroesophageal Reflux Disease (GERD) Chronic low back pain	Lisinopril Pravastatin Pantoprazole Hydrocodone/Acetaminophen	Penicillin	Father–Heart attack Mother–Breast cancer
**Physical Examination**			
**General**	Well-developed, morbidly obese male, in no acute distress.		
**Head, Eyes, Ears, Nose, and Throat (HEENT)**	Normocephalic and atraumatic. Pupils, equal, round, reactive to light and accommodation (PERRLA). Mucosa is pink and moist. Thick facial hair.		
**Neck**	Short and large circumference. Appears generally full due to the goiter.		
**Lungs**	Clear to auscultation bilaterally.		
**Cardiovascular**	Regular rate and rhythm. No murmurs, rubs, or gallops.		
**Abdomen**	Soft and nontender. Normal bowel sounds.		
**Neurological**	Awake, alert, and mostly oriented to person, place, and time. CN II-XII are grossly intact and there are no focal deficits.		
**Skin**	Warm, dry and intact without rashes or lesions.		
**Genitourinary (GU)**	No external masses or lesions.		
**Psychiatric**	Appropriate mood and affect. No visual or auditory hallucinations. No suicidal or homicidal ideation.		

**Table A3:** Instructor Notes–Changes and Case Branch Points.

Intervention/Time point	Change in Case	Additional Information
Initiation of scenario	The leaving anesthesiologist changes over to the new anesthesiologist and walks out.	“This patient was difficult to mask ventilate due to the thick beards and the big tongue” “He was a difficult airway with Mac 3 direct laryngoscope, so we intubated with a video laryngoscope”. “The cuff started to leak during the case, but it resolved after adding another 10 mL of air to the cuff”. “The surgeon requested no paralytics during the case”.
4 min into the scenario	An apparent cuff leak recurs, and the sound is heard inside the patient’s oral cavity. The anesthesia machine may register an air leak from the circuit.	This cuff leak will be simulated by surgeon actor(s) deliberately pushing the endotracheal tube slightly out into the oral cavity with a Kelly forceps.
5 min into the scenario	The anesthesiologist adds more air to the cuff to seal the leak.	The surgeon(s) should deny if the anesthesiologist asks if they have breached the airway.
7 min into the scenario	The endotracheal tube completely dislodges from the glottis, causing complete loss of airway.	This will be simulated by surgeon actor(s) deliberately pushing the endotracheal tube completely out into the oral cavity with a Kelly forceps.
Anesthesiologist attempts bag-mask ventilation	Bag-mask ventilation fails due to thick beards and the obstruction caused by the big tongue.	The mannequin is programmed as maximum tongue swelling. BP 178/92, HR 108, SpO_2_ drops to 70% in 40 s after the loss of airway.
Anesthesiologist restores ventilation by inserting a supraglottic airway	Adequate tidal volume has been achieved and the patient’s oxygenation starts to improve.	BP 149/81, HR 93, SpO_2_ increases to 99% in 30 s after the restoration of ventilation.
Anesthesiologist prepares for endotracheal reintubation with a video laryngoscope	Anesthesiologist draws up propofol and succinylcholine. Sets up the video laryngoscope. May ask the surgeon to remove the shoulder roll.	Surgeon: “Please make sure you don’t use long-acting muscle relaxants”.
Anesthesiologist reintubates the patient	A secure airway has been established.	BP 142/76, HR 81, SpO_2_ 99%
Anesthesiologist tells the surgeon he/she can resume the procedure		This will end the scenario.

**Table A4:** Instructor Notes–Ideal Scenario Flow and Anticipated Management Mistakes.

Ideal Scenario Flow
The learner anesthesiologist walks into the OR. There is a thyroidectomy case already in progress. The other anesthesiologist who started the case changes over the information to the learner anesthesiologist and leaves the OR. The learner anesthesiologist has been informed during the hand-off that the patient is morbidly obese, was a known difficult airway, and required to add extra cuff air to remedy intraoperative cuff leak. A few minutes after taking over the case, the anesthesiologist notices the cuff leak has started again. The surgeon denies damaging the airway. As the anesthesiologist tries to troubleshoot the cuff leak by adding more cuff air or examining the endotracheal tube, the endotracheal tube completely slides out of the patient’s glottis and the complete loss of airway happens. The anesthesiologist immediately notifies the surgeon and the surgery staff in the OR, removes the dislodged endotracheal tube, suctions the oral cavity to clear secretions, then initiates bag-mask ventilation. The bag-mask ventilation fails due to thick facial hair and a large tongue. As the patient’s SpO_2_ starts to drop, the anesthesiologist successfully inserts a supraglottic airway to temporarily restore the ventilation. The SpO_2_ improves and the anesthesiologist prepares for reintubation with propofol, succinylcholine, and a video laryngoscope. After the successful reintubation, the anesthesiologist communicates with the surgeon that he/she can resume the procedure.
**Anticipated Management Mistakes**
**Failure to recognize the accidental extubation:** The anesthesiologist cannot identify the source of the massive air leak after the accidental extubation. He/she may keep thinking the leak is from the anesthesia circuit or the anesthesia machine. Disconnecting the anesthesia circuit from the patient at the Y-piece and performing a quick leak test to confirm there is no gross leak or obstruction in the circuit takes only seconds, and it will indicate the problem is on the patient side. **Failure to restore ventilation with a supraglottic airway:** The anesthesiologist may be fixated on either attempting mask ventilation or too busy preparing to reintubate while the patient is quickly desaturating to a critical level. When the patient is difficult to mask ventilate and already desaturating, the anesthesiologist should immediately secure the airway with a supraglottic airway to restore ventilation. **Failure to administer additional anesthetics upon reintubation:** Since this is an emergency reintubation in the middle of the case, and the patient is already unconscious, the anesthesiologist may forget that additional propofol and muscle relaxation are needed. The anesthesiologist should be reminded that a short-acting muscle relaxant, such as succinylcholine, has been requested by the surgeon. **Failure to utilize a video laryngoscope for the reintubation:** This patient is a known difficult airway and will tend to desaturate relatively quickly. The anesthesiologist should utilize a video laryngoscope to quickly reintubate this patient. If the anesthesiologist fails to reintubate with or without utilizing a video laryngoscope, the patient will continue to desaturate. The anesthesiologist should revert back to supraglottic airway ventilation and ask for extra help or consider aborting the surgery.

## Appendix B. Critical Action Checklist

	**Critical Action**	**Definitely Completed**	**Maybe**	**Missed**
1	Take over the ongoing thyroidectomy case from another anesthesiologist. Acknowledge the critical information regarding the patient’s difficult airway mentioned during the hand-off			
2	Recognize the recurrence of cuff air leak and take action to fix the problem			
3	Recognize the ventilator alarms indicating a complete loss of airway			
4	Identify the cause of the complete loss of airway as accidental extubation			
5	Alert the surgeon and the surgical team in the OR			
6	Remove the extubated endotracheal tube, shut off the vaporizer for the volatile anesthetic, clean the patient’s airway of secretions, and attempt bag-mask ventilation			
7	Ask the surgeon to remove the shoulder roll if necessary			
8	Recognize the quick desaturation of the patient			
9	Ask for more help or call Code Blue			
10	After failing the attempt of bag-mask ventilation, insert a supraglottic airway to restore the ventilation temporarily			
11	Prepare necessary medications for reintubation			
12	Reintubate the patient with a video laryngoscope			
13	Communicate with the surgeon that the airway has been re-established and the patient is ready to resume the surgery			

## Appendix C. Debriefing Materials

**The following points should be discussed during the debriefing with the learners.** 1. **Communication between the anesthesiologists leaving and taking over:** It is a very common practice to sign out an ongoing case to another anesthesiologist. Because the handover takes place in a busy operating room setting, it is common for there to be frequent interruptions. The learner anesthesiologist should discuss how to perform handoffs efficiently and without omissions. Were there any omissions of any critical information during the changeover? What cause did the anesthesiologist taking over assume when he was informed that there was a cuff leak resolved by adding 10 mL of air? Did both anesthesiologists attempt to discuss or explore the root cause? 2. **The cause of accidental extubation:** The facilitator will point out the three factors which contributed to the migration of the endotracheal tube resulting in the accidental extubation. The learners should discuss and understand each mechanism described below. - **Maximum cervical extension:** Although the endotracheal tube has initially been taped at the correct depth, it was pulled out with cervical extension, and the cuff partially herniated out of the vocal cords enough to create an air leak. - **Adding extra cuff air in an attempt to seal the air leak:** As the cuff air was further added to remedy the leak without repositioning the endotracheal tube, the tube continued to slide out although the air leak appeared to have temporarily resolved. Only the distal end of the endotracheal tube remained beyond the vocal cords while the cuff was inflated with more than 20 mL of air at this point. - **Surgical manipulation of the neck:** The vibration and movement transferred from the surgical manipulation of the neck eventually dislodged the endotracheal tube completely out from the vocal cords. 3. **Recognize accidental extubation:** The learners should discuss what alarms they received when the integrity of the airway has been lost. What steps should the learner take to isolate the problem and discover the accidental extubation? 4. **Communication with the surgical team:** Emphasize the importance of notifying the loss of airway and asking for help. Should we immediately activate Code Blue in this situation? 5. **Re-establishment of the secure airway:** The learners should discuss the steps and the potential obstacles in re-establishing the ventilation on a quickly de-saturating, known difficult-airway patient. - **Initiate bag-mask ventilation:** Obstacles–secretions, facial hairs, large tongue contributing to obstructive sleep apnea, maximum cervical extension, head of the OR table being turned away from the anesthesia machine - **Reestablish temporary ventilation with supraglottic airway:** Obstacles–unavailability of the device, wrong device size, large leak from poor seal - **Draw up medicines required for reintubation:** Obstacles–extra medicine not immediately available in the drug tray/OR, nobody to help draw up the medicine - **Reintubate:** Obstacles–known difficult airway, maximum cervical extension, unavailability of video laryngoscope

## Appendix D. Information for the “Anesthesiologist Who Is Signing Out”

**Your role**: You are the Anesthesiologist who is signing out to the new anesthesiologist. You have started the case. During the hand-off, you will mention the following information: 1. The patient is morbidly obese (BMI 53). 2. The patient was a difficult mask ventilation due to thick facial hair and a big tongue. 3. The patient was a known difficult airway, and you used a video laryngoscope to intubate. 4. The endotracheal tube was taped 22 cm at the lip. 5. After the case started, some minor cuff leak started to be heard from the patient’s oral cavity, but the issue has been resolved after adding another 10 mL of air to the cuff.
Mr. Nick McNoneck, a 51-year-old male is undergoing a **total thyroidectomy** for his thyroid goiter. The patient is morbidly obese and known for having a difficult airway. The learner anesthesiologist takes over the case from the other anesthesiologist. Shortly after taking over the case, the endotracheal tube slides out, causing complete loss of airway to occur. The patient quickly starts to desaturate, and the ventilation needs to be re-established as soon as possible. **Allergies:** Penicillin **Past Medical History:** Morbid obesity (BMI 53) Hypertension Hyperlipidemia Obstructive Sleep Apnea GERD Chronic low back pain **Past Surgical History:** Left knee arthroscopy **Current Medications:** Lisinopril Pravastatin Pantoprazole Hydrocodone/Acetaminophen **Social History:** Tobacco Use: 1 pack of cigarettes per day Alcohol Use: socially Illicit Drug Use: Denies **Pertinent Physical Examination:** Height: 186 cm, Weight: 183 kg Vital Signs: BP 127/83 mmHg, HR 76, Sinus rhythm, RR 10 on Volume Controlled Ventilation, SpO_2_ 97% on FiO_2_ 40%, T 36.8 °C General: Well-developed, morbidly obese adult. The patient has been already intubated and under general anesthesia.

## Appendix E. Information for the “Surgeon”

**Your role**: You are the surgeon. You are already scrubbed in for the total thyroidectomy case when the learner anesthesiologist enters the OR. The patient mannequin has a shoulder roll, and the neck has been fully extended for surgical exposure. The scenario involves accidental intraoperative extubation leading to complete loss of airway. In order to reproduce this incident, you will use Kelly forceps to grab the endotracheal tube through the access window of the mannequin’s neck and gradually push the tube out into the oral cavity. Since this scenario is NOT intended to reproduce a surgical breach of the airway, you must deny if the anesthesiologist questions if you have injured the trachea or the endotracheal tube. You also have to block the view of the surgical field from the anesthesiologist so that he/she cannot see your manipulation of the airway. Once the anesthesiologist recognizes the loss of airway, communicate with he/she and offer help. Do request “not to use any longer acting muscle relaxant” as the anesthesiologist attempts to reintubate, since you will need to monitor the recurrent laryngeal nerve during the surgery.
Mr. Nick McNoneck, a 51-year-old male is undergoing a total thyroidectomy for his thyroid goiter. The patient is morbidly obese and known for having a difficult airway. The learner anesthesiologist takes over the case from the other anesthesiologist. Shortly after taking over the case, the endotracheal tube slides out, causing complete loss of airway to occur. The patient quickly starts to desaturate, and the ventilation needs to be re-established as soon as possible. **Allergies:** Penicillin **Past Medical History:** Morbid obesity (BMI 53) Hypertension Hyperlipidemia Obstructive Sleep Apnea GERD Chronic low back pain **Past Surgical History:** Left knee arthroscopy **Current Medications:** Lisinopril Pravastatin Pantoprazole Hydrocodone/Acetaminophen **Social History:** Tobacco Use: 1 pack of cigarettes per day Alcohol Use: socially Illicit Drug Use: Denies **Pertinent Physical Examination:** Height: 186 cm, Weight: 183 kg Vital Signs: BP 127/83 mmHg, HR 76, Sinus rhythm, RR 10 on Volume Controlled Ventilation, SpO_2_ 97% on FiO_2_ 40%, T 36.8 °C General: Well-developed, morbidly obese adult. The patient has been already intubated and under general anesthesia.
